# Pathway-gene identification for pancreatic cancer survival via doubly regularized Cox regression

**DOI:** 10.1186/1752-0509-8-S1-S3

**Published:** 2014-01-24

**Authors:** Haijun Gong, Tong Tong Wu, Edmund M Clarke

**Affiliations:** 1 Department of Mathematics and Computer Science, Saint Louis University, Saint Louis, MO 63103, USA; 2 Department of Biostatistics and Computational Biology, University of Rochester, Rochester, NY 14642, USA; 3 Computer Science Department, Carnegie Mellon University, Pittsburgh, PA 15213, USA

## Abstract

**Background:**

Recent global genomic analyses identified 69 gene sets and 12 core signaling pathways genetically altered in pancreatic cancer, which is a highly malignant disease. A comprehensive understanding of the genetic signatures and signaling pathways that are directly correlated to pancreatic cancer survival will help cancer researchers to develop effective multi-gene targeted, personalized therapies for the pancreatic cancer patients at different stages. A previous work that applied a LASSO penalized regression method, which only considered individual genetic effects, identified 12 genes associated with pancreatic cancer survival.

**Results:**

In this work, we integrate pathway information into pancreatic cancer survival analysis. We introduce and apply a doubly regularized Cox regression model to identify both genes and signaling pathways related to pancreatic cancer survival.

**Conclusions:**

Four signaling pathways, including Ion transport, immune phagocytosis, TGF*β *(spermatogenesis), regulation of DNA-dependent transcription pathways, and 15 genes within the four pathways are identified and verified to be directly correlated to pancreatic cancer survival. Our findings can help cancer researchers design new strategies for the early detection and diagnosis of pancreatic cancer.

## Background

Pancreatic cancer [1] is a devastating disease with a very poor prognosis and a five-year survival rate around 3-5%. The most common form of pancreatic cancer is the pancreatic ductal adenocarcinoma (PDAC, a malignant exocrine cancer). In the past 30 years, no substantial progress has been made in PDAC diagnosis and treatment. New techniques and methods to investigate the dynamics of PDAC are urgently needed. Modern microarray technology has revolutionized the way that we study the complex biological systems, allowing pancreatic cancer researchers to make genome-wide expression profiling and measure other features for patients in a fast, precise, and cost-effective way. One aim of systems biologists is to correctly decipher and interpret the high-dimensional complex gene expression data, that is, to identify the key genetic signatures and signaling pathways implicated in the diseases.

Pancreatic cancer is characterized by rapid growth, early local and distant invasion, interactions with stromal cells (e.g., pancreatic stellate cells) [2] and fibrous tissue, and a high resistance to chemotherapy and radiotherapy. The evolution of pancreatic cancer is partially stimulated by the overexpression of several growth factors, cytokines, and genetic alterations [3,4] at different stages of PDAC. Recent global genomic analyses identified 69 gene sets and 12 core signaling pathways genetically altered in the pancreatic cancer [1]. Most of the previous genomic analyses and microarray studies focused on the identification of the differentially expressed and metastasis-associated genes at different stages of pancreatic cancer [3,5], ignoring an important clinical factor - survival time. Stratford et al.'s work identified six genetic signatures [6] associated with metastatic pancreatic cancer using a sequence of statistical techniques, including the significance analysis of microarray (SAM) [7], centroid-based predictor [8], Pearson correlation, X-Tile [9], Kaplan-Meier estimator [10] and Cox model [11]. Though these genes could help discriminate high- and low-risk patients, the prediction was not based on survival time. A comprehensive understanding of the genetic signatures and signaling pathways that are directly correlated to pancreatic cancer survival will help cancer researchers to develop effective multi-gene targeted, personalized therapies for the pancreatic cancer patients at different stages and improve survival rate.

The Cox proportional hazards model [11] is the most popular survival model used to describe the relationship between the patient's survival time and predictor variables. When we have high-dimensional data (e.g. in a microarray study) where the number of predictors (genes) far exceeds the number of subjects (patients), the Cox model cannot be fitted directly unless the high-dimensionality is properly handled. The regularization approach has been widely used to select important variables from a large pool of candidate variables [12-14]. For example, a Lasso (least absolute shrinkage and selection operator) penalty can be imposed to individual variables to automatically remove unimportant ones by shrinking their regression coefficients to be exactly zero [15]. In our previous work [16], we applied a lasso penalized Cox regression method, for the first time, to investigate the signature genes that are correlated to the pancreatic cancer survival time. We identified 12 genes associated with the pancreatic cancer survival and eight of them have been confirmed to be genetically altered and differentially expressed in the cancer of gastric, colorectal, ovarian, breast, skin, kidney, colon, lung, and pancreatic in *in vivo *and *in vitro *experiments [17-25]. It has been shown that these survival-associated genes can also help to grade the stage and estimate the survival time of the PDAC patients.

However, the genes may perform as groups rather than individuals since some genes belong to the same pathways and get involved in the same biological processes. The pathway information is biologically important to our understanding of gene regulatory networks and cancer development [1]. The previous work [16] performs gene selection based on the strength of individual genes solely and ignores the information of signaling pathways. Recently, several variable selection methods have been introduced to consider group effects. For example, the group lasso method penalizes the *L*_2_-norm (Euclidean norm) of the coefficients within each group in linear regression [26] and Cox proportional hazards model [12]. Based on the boosting technique, a group additive regression model [27] and a nonparametric pathway-based regression model [28] were developed to identify groups of genomic features that are related to several clinical phenotypes, including the survival outcome. However, those group selection methods only conduct "group selection" without "within-group selection", since they select variables in an "all-in-or-all-out" fashion. That is, if one variable in a group is selected, all the other variables in the same group will also get selected.

Although pathways as a whole are involved in the development of pancreatic cancer, according to the global genomic analyses, not all the genes in the same pathway are involved in the process. In this work, we employ a doubly regularized Cox (DrCox) regression model [29] that integrates both genes and signaling pathways for the pancreatic cancer survival analysis. Both non-overlap and overlap cases of DrCox are considered. Cyclic coordinate descent algorithms are derived for parameter estimation. We analyze the high-dimensional microarray data of pancreatic cancer patients with localized and resected PDAC collected between 1999 and 2007 [6] using DrCox. Four signaling pathways, including Ion transport, immune phagocytosis, TGF*β *(spermatogenesis), regulation of DNA-dependent transcription pathways, and 15 genes within these four pathways are identified and verified to be directly correlated to pancreatic cancer survival. Compared with other methods, the DrCox model can provide more accurate and useful prediction of survival time [29]. These findings can help cancer researchers design new strategies for the early detection and diagnosis of pancreatic cancer at different stages.

## Methods

In this section, we describe the doubly regularized Cox (DrCox) regression and derive the parameter estimates via cyclic coordinate descent algorithms. We first present the case where the groups do not overlap, i.e., each variable belongs to only one group. Then we discuss the overlap case, i.e., variables are allowed to belong to multiple groups.

### Doubly regularized Cox (DrCox) regression for non-overlap cases

Assume that the *p *variables (genes) occur in *K *groups (pathways). We further assume the *k*th group has *p_k _*variables and denote the *p_k _*variables in the *k*th group by ***X***_(*k*) _= (*X*_*k*1_, . . . , *X*_*kpk*_)*^T^*, with the corresponding regression coefficients ***β***_(*k*) _= (*β*_*k*1_, . . . , *β_kpk_*)*^T^*. For a sample of *n *subjects, let *T_i _*and *C_i _*denote the survival time and the censoring time for subject *i *= 1, . . . , *n*. The observed survival time is defined by *Y_i _*= min{*T_i_*, *C_i_*} and the censoring indicator is *δ_i _*= ***I***(*T_i _*≤ *C_i_*). The *p *predictor variables of the *i*th subject is denoted by $X_{i}={\left(X_{i,\left(1\right)}^{T},\;\ldots ,\;X_{i,\left(K\right)}^{T}\right)}^{T}$, where $X_{i,\left(k\right)}={\left(X_{i,k1},\;\ldots ,\;X_{i,kp_{k}}\right)}^{T}$. The survival time *T_i _*and the censoring time *C_i _*are conditionally independent given ***X***_*i*_. The censoring mechanism is assumed to be noninformative. The observed data can be represented by the triplets {(*Y_i_*, *δ_i_*, ***X**_i_*), *i *= 1, . . . , *n*}.

The Cox proportional hazards model [11] composed of *p *genes and *K *pathways is written by

$$h\left(t|X\right)=h_{0}\left(t\right)\text{exp}\left(\overset{K}{\underset{k=1}{\sum }}\overset{p_{k}}{\underset{j=1}{\sum }}\beta_{kj}X_{kj}\right)=h_{0}\left(t\right)\text{exp}\left(\overset{K}{\underset{k=1}{\sum }}X_{i,\left(k\right)}^{T}\beta_{\left(k\right)}\right),$$

where $\sum_{k=1}^{K}p_{k}=p$. The partial likelihood of the Cox model is

$$L_{n}\left(\beta \right)=\underset{i\in D}{\prod }\frac{\text{exp}\left(\sum_{k=1}^{K}X_{i,\left(k\right)}^{T}\beta_{\left(k\right)}\right)}{\sum_{l\in R_{i}}\text{exp}\left(\sum_{k=1}^{K}X_{l,\left(k\right)}\beta_{\left(k\right)}\right)},$$

where *D *is the set of indices of observed failures, and *R_i _*is the set of indices of the subjects who are at risk at time *Y_i_*.

To achieve the goal of both group and within-group variable selection and to overcome the non-convexity drawback, the doubly regularized Cox regression model imposes a mixture of lasso penalty and group lasso penalty to the log-partial likelihood *ℓ_n_*(***β***) = log *L_n_*(***β***)

(1) $$\begin{array}{c} g\left(\beta \right)\;=\;-\ell_{n}\left(\beta \right)+\lambda_{1}\overset{K}{\underset{k=1}{\sum }}\overset{p_{k}}{\underset{j=1}{\sum }}|\beta_{kj}|+\lambda_{2}\overset{K}{\underset{k=1}{\sum }}\sqrt{\overset{p_{k}}{\underset{j=1}{\sum }}\beta_{kj}^{2}}\\ \;\;\;\;\;\;\;\;\;\;\;\;\;\;\;\;=-\ell_{n}\left(\beta \right)+\lambda_{1}\overset{K}{\underset{k=1}{\sum }}∥\beta_{\left(k\right)}{∥}_{1}+\lambda_{2}\overset{K}{\underset{k=1}{\sum }}∥\beta_{\left(k\right)}{∥}_{2}, \end{array}$$

where $∥\beta_{\left(k\right)}{∥}_{1}=\sum_{j=1}^{px}|\beta_{kj}|$ is the lasso penalty on individual parameters, $∥\beta_{\left(k\right)}{∥}_{2}=\sqrt{\sum_{j=1}^{p_{k}}\beta_{kj}^{2}}$ is the group penalty on groups of parameters, and *λ*_1 _and *λ*_2 _are two nonnegative tuning constants controlling the strength of variables selection. The larger are the tuning constants, the fewer variables are retained in the model. In this paper, the value of the tuning constants are determined using *k*-fold cross validation (data-driven) technique to select a subset of relevant genes and signaling pathways for accurate and robust prediction.

### Coordinate descent for non-overlap cases

Since there are more predictor variables than subjects (*p *>*n*), to tackle the high-dimensionality problem we use a cyclic coordinate descent algorithm, which has been shown to be computationally efficient [30-33]. The idea is to break a large optimization problem into a sequence of small ones. In other words, instead of estimating all the parameters at the same time, we can update each parameter one by one. Readers can refer to [31,32] for more details.

In the non-overlap case, where each variable belongs to only one group, estimation of parameters and selection of important variables can be conducted via the minimization of (1) iteratively w.r.t. one parameter by one parameter. The first step is to calculate the forward and backward directional derivatives of each parameter. If *e_kj _*is the coordinate direction along which *β_kj _*varies, then the forward and backward directional derivatives of *β_kj _*are

$$\begin{array}{c} d_{e_{kj}}g\left(\beta \right)\;=\;\underset{t↓0}{\text{lim}}\frac{g\left(\beta +te_{kj}\right)-g\left(\beta \right)}{t}\\ =-\frac{\partial }{\partial \beta_{kj}}\ell_{n}\left(\beta \right)+\left\{\begin{array}{cc} \;\left(\lambda_{1}+\lambda_{2}\right){\left(-1\right)}^{I\left(\beta_{kj}<0\right)} & \text{if}\;∥\beta_{\left(k\right)}{∥}_{2}=0\\ \;\lambda_{1}{\left(-1\right)}^{I\left(\beta_{kj}<0\right)}+\lambda_{2}\frac{\beta_{kj}}{∥\beta_{\left(k\right)}{∥}_{2}} & \text{if}\;∥\beta_{\left(k\right)}{∥}_{2}>0, \end{array}\right) \end{array}$$

and

$$\begin{array}{c} d_{-e_{kj}}g\left(\beta \right)\;=\;\underset{t↓0}{\text{lim}}\frac{g\left(\beta -te_{kj}\right)-g\left(\beta \right)}{t}\\ \;\;\;\;\;\;=\frac{\partial }{\partial \beta_{kj}}\ell_{n}\left(\beta \right)+\left\{\begin{array}{cc} \;\left(\lambda_{1}+\lambda_{2}\right){\left(-1\right)}^{I\left(\beta_{kj}>0\right)} & \text{if}\;∥\beta_{\left(k\right)}{∥}_{2}=0\\ \;\lambda_{1}{\left(-1\right)}^{I\left(\beta_{kj}>0\right)}-\lambda_{2}\frac{\beta_{kj}}{∥\beta_{\left(k\right)}{∥}_{2}} & \text{if}\;∥\beta_{\left(k\right)}{∥}_{2}>0, \end{array}\right) \end{array}$$

where *I*(*·*) is an indicator function equal to 1 if the condition in the parentheses is satisfied and 0 otherwise, and

$$\frac{\partial }{\partial \beta_{kj}}\ell_{n}(\beta )=\sum_{i\in D}\{x_{i,kj}-\frac{\sum_{l\in R_{i}}\exp\left(\sum_{k=1}^{K}X_{l,(k)}^{T}\beta_{\left(k\right)}\right)x_{l,kj}}{\sum_{l\in R_{i}}\exp\left(\sum_{k=1}^{K}X_{l,(k)}^{T}\beta_{\left(k\right)}\right)}\}.$$

After obtaining the directional derivatives, we then need to decide which parameters to be updated and the direction for updating. If both of the directional derivatives $d_{e_{kj}}g\left(\beta \right)$ and $d_{-e_{kj}}g\left(\beta \right)$ are nonnegative, then the update for *β_kj _*is skipped. If either directional derivative is negative, then we solve for the minimum along the corresponding direction. It is impossible for both directional derivatives to be negative due to the convexity of *g*(***β***). After identifying the direction to update the parameter, one can use Newton's method to solve for the minimum. The update at iteration *m *+ 1 is given by

$$\beta_{kj}^{m+1}\;=\;\beta_{kj}^{m}+\frac{-\frac{\partial }{\partial \beta_{kj}}\ell_{n}\left(\beta^{m}\right)+\lambda_{1}{\left(-1\right)}^{I\left(\beta_{kj}^{m}<0\right)}}{\frac{\partial^{2}}{\partial \beta_{kj}^{2}}\ell_{n}\left(\beta^{m}\right)}+\frac{\lambda_{2}\left\{{\left(-1\right)}^{I\left(\beta_{kj}^{m}<0\right)}I_{1}\left(\beta_{\left(k\right)}^{m}\right)+\frac{\beta_{kj}^{m}}{∥\beta_{\left(k\right)}^{m}{∥}_{2}}I_{2}\left(\beta_{\left(k\right)}^{m}\right)\right\}}{\frac{\partial^{2}}{\partial \beta_{kj}^{2}}\ell_{n}\left(\beta^{m}\right)}$$

where ***β****m *is the estimate at iteration *m*, *I*_1_(*·*) = *I*(ǁ *· ǁ*_2 _= 0), and *I*_2_(*·*) = *I*(ǁ *· ǁ*_2 _*>*0).

### DrCox regression via coordinate descent for overlap cases

However, in reality, one gene can get involved in different pathways. To consider overlapping, we modify the notation and objective function (1). We denote the *p *variables by *X*_1_, . . . , *X_p _*with the corresponding regression coefficients *β*_1_, . . . , *β_p_*. Let *V_k _*⊆ {1, 2, . . . , *p*} be the set of indices of variables in the *k*th group. The objective function designed for the overlap case can be written as

(2) $$g\left(\beta \right)=-\ell_{n}\left(\beta \right)+\lambda_{1}\overset{p}{\underset{j=1}{\sum }}|\beta_{j}|+\lambda_{2}\overset{K}{\underset{k=1}{\sum }}\sqrt{\underset{j\in V_{k}}{\sum }\beta_{j}^{2}}.$$

Note that predictor *X_j _*can belong to several pathways but it is only associated with one coefficient *β_j_*.

The parameter estimation needs to be modified accordingly. If we consider the coordinate direction *e_j _*for *β_j_*, the forward and backward directional derivatives of *β_j _*are

$$\begin{array}{c} d_{e_{j}}g\left(\beta \right)\;=\;\underset{t↓0}{\text{lim}}\frac{g\left(\beta +te_{j}\right)-g\left(\beta \right)}{t}\\ =\;-d_{e_{j}}\ell_{n}\left(\beta \right)+\lambda_{1}{\left(-1\right)}^{I\left(\beta_{j}<0\right)}+\lambda_{2}\underset{k\in G_{j}}{\sum }\left\{{\left(-1\right)}^{I\left(\beta_{j}<0\right)}I_{1}\left(\beta_{\left(k\right)}\right)+\frac{\beta_{j}}{∥\beta_{\left(k\right)}{∥}_{2}}I_{2}\left(\beta_{\left(k\right)}\right)\right\} \end{array}$$

and

$$\begin{array}{c} d_{-e_{j}}g\left(\beta \right)=\underset{t↓0}{\text{lim}}\frac{g\left(\beta -te_{j}\right)-g\left(\beta \right)}{t}\\ =\;-d_{-e_{j}}\ell_{n}\left(\beta \right)+\lambda_{1}{\left(-1\right)}^{I\left(\beta_{j}>0\right)}+\lambda_{2}\underset{k\in G_{j}}{\sum }\left\{{\left(-1\right)}^{I\left(\beta_{j}>0\right)}I_{1}\left(\beta_{\left(k\right)}\right)-\frac{\beta_{j}}{∥\beta_{\left(k\right)}{∥}_{2}}I_{2}\left(\beta_{\left(k\right)}\right)\right\}. \end{array}$$

where *G_j _*⊆ {1, 2, . . . , *K*} are the indices of groups that *X_j _*belongs to. After determining the direction for updating, the coefficient can be updated by

$$\beta_{j}^{m+1}=\beta_{j}^{m}+\frac{-\frac{\partial }{\partial \beta_{j}}\ell_{n}\left(\beta^{m}\right)+\lambda_{1}{\left(-1\right)}^{I\left(\beta_{j}^{m}<0\right)}}{\frac{\partial^{2}}{\partial \beta_{j}^{2}}\ell_{n}\left(\beta^{m}\right)}+\frac{\lambda_{2}\sum_{k\in G_{j}}\left\{{\left(-1\right)}^{I\left(\beta_{j}^{m}<0\right)}I_{1}\left(\beta_{\left(k\right)}^{m}\right)+\frac{\beta_{j}^{m}}{∥\beta_{\left(k\right)}^{m}{∥}_{2}}I_{2}\left(\beta_{\left(k\right)}^{m}\right)\right\}}{\frac{\partial^{2}}{\partial \beta_{j}^{2}}\ell_{n}\left(\beta^{m}\right)}.$$

## Results and discussion

**Figure 1:**
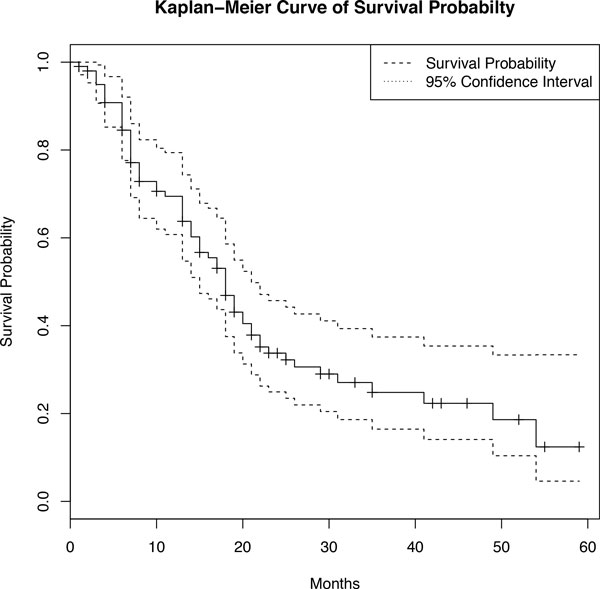
**Kaplan-Meier curve of survival probability and 95% confidence interval for 102 PDAC patients** The Kaplan-Meier survival curve (solid line) describes the probability of survival for the 102 PDAC patients. The dashed lines represent the 95% confidence interval. The horizontal axis represents the survival time (in months). Each step means an actual event happens, i.e. a pancreatic cancer patient dies. A short vertical line without a drop means a patient get censored for different reasons, e.g. drops off the study or the study ends.

The DrCox model with the cyclic coordinate descent algorithm is applied to analyze the PDAC data collected between 1999 and 2007. The aim of this work is to identify core signaling pathway sets and genetic signatures within those pathways related to pancreatic cancer survival. The microarray data of pancreatic cancer include 102 samples [6], which are publicly available at Gene Expression Omnibus (access code 21501). According to [6], among these 102 PDAC patients, 66 died at the end of the study (censoring rate 35%). The survival time ranges from 1 month to 5 years. The Kaplan-Meier curve is plotted in Figure 1 to show the probability of survival in 5 years for the 102 PDAC patients. Each step means an actual event happens, i.e. a pancreatic cancer patient dies. A short vertical line without a drop means a patient gets censored for different reasons, drops off the study or the study ends. Additionally, two stage variables, T stage and N stage, are given to describe the stages of pancreatic cancer, where T stage describes the size of the primary tumor ranging from 1 to 4 and N stage describes the spread to nearby (regional) lymph nodes with values 0 or 1.

**Figure 2:**
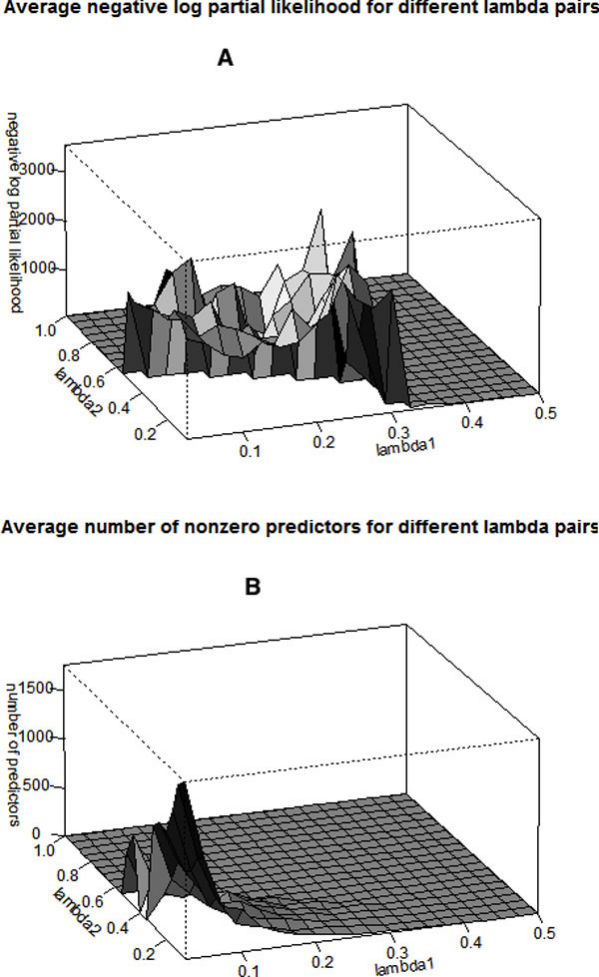
**3-fold cross validation for tuning constants *(*λ*_1_*, λ*_2_)***. (A) shows the 3-D plot of the log-partial likelihood function vs. (λ_1_, λ_2_), and the optimal values are λ_1 _= 0.3 and λ_2 _= 0.1; (B) shows the 3-D plot of the number of selected genes with nonzero regression coefficients vs. (λ_1_, λ_2_).

The whole dataset is randomly split into the training, validation, and testing sets with equal sizes. The training set is used for model fitting, and the validation set is used for tuning constants selection. Using the 3-fold cross-validation, we got the optimal values of *λ*_1 _= 0.3 and *λ*_2 _= 0.1, which minimize the log-partial likelihood function. Figure 2 shows the 3-D plots of the log-partial likelihood function and the number of selected genes vs. (*λ*_1_, *λ*_2_), respectively. Under the optimal tuning constants, 4 pathways and 15 genes are selected from the pool of 12660 probes of 6910 genes in 130 pathway sets organized in [1], which belong to 15 core groups in the pancreatic cancer studies. The selected pathways include the pathways of "regulation of DNA-dependent transcription" (6 out of 2096 genes are selected), "Ion transport" (7 out of 555 genes are selected), "immune phagocytosis" (1 out of 215 genes is selected), and "TGF*β *(spermatogenesis)" (1 out of 268 genes is selected) pathways. These identified pathways and genes are biologically meaningful and consistent with the existing scientific findings. In particular, three genes - ZNF233, SLC22A8, and PCYT1B - were identified in the previous work [16] using a Lasso penalized Cox model when considering gene signatures only.

• **Regulation of DNA-dependent transcription pathway **is well-known to be related to the development of cancer. It regulates the frequency and rate of cellular DNA-dependent transcription. This work identified three families of six genes that are related to pancreatic cancer survival. The six genes are DENND4A, KLF13, ZNF229, ZNF233, ZNF395, and ZNF432.

- **DENND4A **is a c-myc promoter-binding protein [34], which mediates signal transduction in the nucleus and regulate the DNA replication and transcription. DENND4A can also activate the RAB10 protein, which is a key regulator of polarized sorting in epithelial cells, from an inactive GDP-bound form to an active GTP-bound form through promoting GDP *→ *GTP exchange.

- **KLF13 **belongs to the KLF family of transcription factors for several oncogenes and tumor suppressor genes [35,36] and it plays an important role in the tumor progression [36]. Recent study shows that KLF13 is overexpressed in the oral cancer cells. Inhibiting KLF13's expression can decrease the proliferation of cancer cell and increase its sensitivity to ionizing radiation [36]. In pancreatic cancer, KLF13 can suppress the cell growth and neoplastic transformation mediated by K-RAS, which is mutated in more than 90% of pancreatic tumors [35]. Our work suggests that KLF13 may be a useful biomarker for early detection and possible targets for the pancreatic cancer therapy.

- **Zinc finger protein family members: ZNF229, ZNF233, ZNF395 and ZNF432 **are DNA-binding protein domains consisting of zinc fingers. Many of these zinc finger proteins, including ZNF233 (also identified in the previous work [16]), have been found to be associated with the abnormality of chromosome 19 in the studies of kidney [23] and pancreatic cancers [1]. Our analysis reveals that Zinc finger proteins and the corresponding pathway might be associated with the survival of pancreatic cancer.

• **Ion transport pathway **plays integral roles in the development of cancer. Since the plasma membrane ion channels contribute to all basic cellular process [37,38], many ion channels are implicated in the uncontrolled proliferation, decreased apoptosis, and unorganized angiogenesis. According to [37], the ion channels also contribute to the six hallmarks of cancer [39]: "1) self-sufficiency in growth signals, 2) insensitivity to antigrowth signals, 3) evasion of programmed cell death (apoptosis), 4) limitless replicative potential, 5) sustained angiogenesis and 6) tissue invasion and metastasis."

We identified seven genes from three different channels or families, including the TRP channel (TRPV5 and TRPM6) regulating the transcellular *Ca*^2+^ transport, KCNK channel (KCNK3 and KCNK18) regulating the *K*^+^ transport, and solute carrier (SLC) family (SLC22A8, SLC8A3, and SLC24A6). Recent experimental studies have indicated that these three families play important roles in the cancer development.

- **TRP (***Ca*^2^+**) channel and TRPV5, TRPM6 genes **regulate the Calcium-mediated signal transduction that is frequently altered in cancer [40]. Several genes in TRPV channel have been detected to be up-regulated in prostate, colon, and breast cancer cells [40-42]. Particularly, TRPV5 and TRPV6 genes exhibit unusually high levels of single nucleotide polymorphisms (SNPs) in African populations as compared to other populations [41]. Moreover, the genes TRPM6 and TRPM7 in the TRPM channel can enhance the secretion of angiogenic factors, for example VEGF [40], resulting in a sustained unorganized angiogenesis process. The TRP channel and TRPV5, TRPM6 genes identified in pancreatic cancer survival data could be possible targets for the future cancer diagnosis and treatment.

- **KCNK (***K*+**) channel and KCNK3, KCNK18 genes **regulate the potassium (*K*+) transport and membrane potential (Vm) in response to different physical and chemical factors [38,40]. Several KCNK channel genes, for example, KCNK9 [43], are overexpressed in breast and lung cancers, and the gene KCNK2 can promote prostate cancer cell's growth [40,44].

- **SLC family: **SLC22A8, SLC8A3, SLC24A6 are membrane transport proteins that are involved in the transport and excretion of many organic ions, drugs and toxicants. Some genes in SLC family are cancer-related, for example, SLC43A2 whose overexpression is associated with the adenocarcinomas and squamous cell carcinoma [45], which was identified in the previous work [16].

• **Immune phagocytosis pathway and CYBA gene: **One prominent hallmark feature of cancer is the evasion of immune destruction [39]. The immune system is important in preventing tumor initiation and controlling tumor growth through identifying and eliminating the cancer cells (i.e., tumor immune surveillance) [46]. Macrophages and other phagocytic cells are important players in the innate immune system whose functions include phagocytosis (homeostatic cell clearance), antigen presentation (pathogen defense), and cytokine production (inflammatory responses). Recent evidence [46-48] revealed that the active immune phagocytosis pathway could inhibit tumor growth through phagocytic clearance, i.e., programmed cell removal in clearing damaged and foreign cells. The CYBA gene is a tumor suppressor [49], which regulates the immune system cells - phagocytes, involved in autophagy. The phagocytosis and superoxide production is primary regulated by the cytochrome b- 245, (light) alpha subunit (also known as *p*22*^phox^*), which is encoded by the gene CYBA. CYBA's mutation will cause the failure of phagocytosis and immune defects [50]. This observation supports our prediction that the immune phagocytosis and tumor suppressor gene CYBA might be associated with pancreatic cancer survival and tumor immune evasion. Targeting this pathway might lead to effective cancer immunotherapies.

• **TGF***β ***core pathway (spermatogenesis signaling set) and PCYT1B gene**: The transforming growth factor beta (TGF*β*) signaling pathway is critical in regulating many cellular processes, including the cell growth, differentiation and apoptosis. It has genetic alterations in 100% of pancreatic cancers [1]. The gene PCYT1B (phosphate cytidylyl transferase 1 choline *β*) was identified to be associated with pancreatic cancer survival, which is consistent with the previous work [16]. The expression of PCYT1B is frequently deregulated in cancer cells of epithelial ovarian [21], high grade gliomas [51], and pancreatic ductal adenocarcinoma [22]. Moreover, PCYT1B is a key regulator in the choline phospholipid metabolism, which is altered in the cancers of breast [19], colon [20], ovarian [21], and gliomas [51]. These observations support our prediction that PCYT1B and TGF*β *pathway are correlated with pancreatic cancer survival and they might help to grade the stage of pancreatic cancer patients.

**Figure 3:**
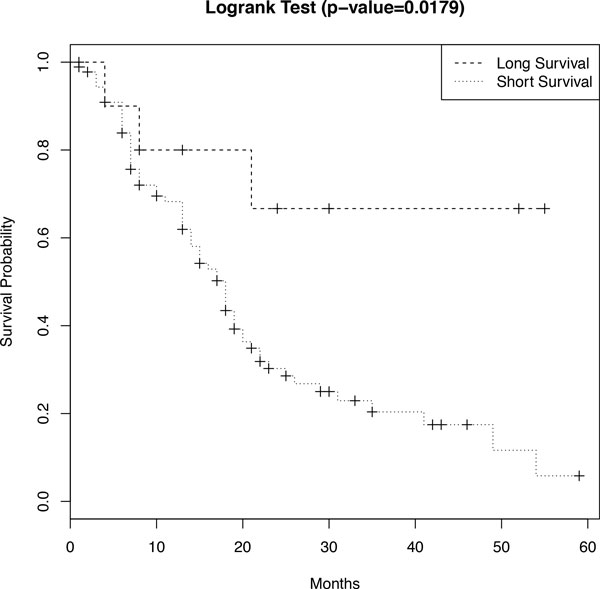
**Logrank test of the long- and short-survival groups based on the 4 pathways and 15 genes (p-value = 0.0179)**. The 102 PDAC patients are divided into long- and short-survival groups based on the 4 pathways and 15 genes. The survival probabilities of these two groups are compared using the logrank test. The p-value of 0.0179 means the two groups are well separated and our finding of 4 pathways and 15 genes is significant.

Compared with the previous work [16], which selected 12 survival-relevant genes using a Lasso penalized Cox model, the DrCox model identified 4 pathways and 15 genes related to pancreatic cancer survival. We divide the patients into long- and short-survival groups based on the selected pathways and genes and conduct the logrank test to compare the two groups. The survival probabilities of these two groups are plotted in the Figure 3. The logrank test gives a p-value of 0.0179, which means the two groups can be well separated and our finding of 4 pathways and 15 genes is significant.

## Conclusions

In this work, we employed the doubly regularized Cox (DrCox) regression coupled with the coordinate descent algorithm to analyze the high-dimensional gene expression data of patients with localized and resected PDAC. Different from the previous work [16], this DrCox model can incorporate both gene and pathway information and simultaneously infer genetic signatures and important signaling pathways that are related to the pancreatic cancer survival. The proposed cyclic coordinate descent algorithm can quickly remove irrelevant genes and signaling pathways, so the prediction of survival time is more accurate and robust than other methods. Other group selection models select variables in an "all-in-or-all-out" fashion with no within-group selection, that is, if one variable in a group (pathway) is selected, all the other variables in the same group will get selected. For example, if gene PCYT1B in the TGF*β *pathway is selected, all the rest of genes in the TGF*β *pathway will be selected as well. However, not all the genes in the TGF*β *pathway are involved in the development of pancreatic cancer. The advantage of our DrCox method is that it can conduct both group selection and within-group selection simultaneously and eliminate the irrelevant.

This work identified four signaling pathways, including Ion transport, immune phagocytosis, TGF*β *(spermatogenesis), regulation of DNA-dependent transcription pathways, and 15 genes within these four pathways, which are directly correlated to pancreatic cancer survival. Pancreatic cancer patients with these deregulated signaling pathways and mutated genes might have a shorter survival time. Several inferred signaling components have been confirmed to be altered frequently in the cancer of pancreatic, oral, prostate, colon, breast and lung in the *in vivo *or *in vitro *experiments. Our finding predicts that, the TRP (*Ca*^2^+) channel-related genes (TRPV5 and TRPM6) and KCNK (*K*+) channel-related genes in the ion transport pathway are possible biomarkers of pancreatic cancer survival. The Immune phagocytosis pathway with the tumor suppressor CYBA gene, which regulates the immune system cells and autophagy through phagocytic clearance, have not received enough attention in the existing pancreatic cancer research literature. The gene PCYT1B in the TGF*β *pathway is frequently deregulated in cancer cells compared with normal cells, which might help to grade the stage of pancreatic cancer patients. The KLF13 in the regulation of DNA-dependent transcription pathway could regulate the cell growth through regulating KRAS pathway. These findings demonstrate that these survival-associated genetic signatures and pathways could be useful biomarkers for early cancer detection and diagnosis and help pancreatic cancer researchers to grade the cancer stage and select appropriate therapies to prolong the patient's survival time at different stages.

This work is the first attempt to infer the pancreatic cancer survival-associated signaling pathway sets and genetic signatures within those pathways using statistical techniques. However, any statistical findings need to be tested by the further clinical and wet lab experiments of pancreatic cancer. We are unable to test our results with other independent datasets in this paper due to the data source limitation. We do expect our results can get verified or falsified by further investigation. We hope the genetic signatures and pathways found in this paper could help cancer researchers design new strategies for the early detection and diagnosis and lead to effective treatments and immunotherapies for pancreatic cancer.

## Competing interests

The authors declare that there are no competing interests.

## Authors' contributions

HG and TTW are joint first authors and both authors contributed equally. HG, TTW, and EMC proposed the study, TTW prepared the computational code, HG and TTW analyzed the results and wrote the manuscript. All authors read and approved the final manuscript.

## Declarations

Publication of this article was funded by the Saint Louis University to HG and NSF grant CCF-0926181 to TTW.

This article has been published as part of *BMC Systems Biology *Volume 8 Supplement 1, 2014: Selected articles from the Twelfth Asia Pacific Bioinformatics Conference (APBC 2014): Systems Biology. The full contents of the supplement are available online at http://www.biomedcentral.com/bmcsystbiol/supplements/8/S1.
